# Evidence-informed policymaking in practice: country-level examples of use of evidence for iCCM policy

**DOI:** 10.1093/heapol/czv033

**Published:** 2015-10-29

**Authors:** Daniela C Rodríguez, Jessica Shearer, Alda RE Mariano, Pamela A Juma, Sarah L Dalglish, Sara Bennett

**Affiliations:** ^1^Johns Hopkins Bloomberg School of Public Health, Dept. of International Health, 615 N Wolfe Street, Baltimore, MD 21205, USA,; ^2^Universidade Eduardo Mondlane, Community Health Department, Maputo, Mozambique and; ^3^African Population and Health Research Center, Nairobi, Kenya

**Keywords:** Case management, child health, evidence-based policy, Kenya, Mozambique, Niger, policy analysis, sub-Saharan Africa

## Abstract

Integrated Community Case Management of Childhood Illness (iCCM) is a policy for providing treatment for malaria, diarrhoea and pneumonia for children below 5 years at the community level, which is generating increasing evidence and support at the global level. As countries move to adopt iCCM, it becomes important to understand how this growing evidence base is viewed and used by national stakeholders. This article explores whether, how and why evidence influenced policy formulation for iCCM in Niger, Kenya and Mozambique, and uses Carol Weiss’ models of research utilization to further explain the use of evidence in these contexts. A documentary review and in-depth stakeholder interviews were conducted as part of retrospective case studies in each study country. Findings indicate that all three countries used national monitoring data to identify the issue of children dying in the community prior to reaching health facilities, whereas international research evidence was used to identify policy options. Nevertheless, policymakers greatly valued local evidence and pilot projects proved critical in advancing iCCM. World Health Organization and United Nations Children's Fund (UNICEF) functioned as knowledge brokers, bringing research evidence and experiences from other countries to the attention of local policymakers as well as sponsoring site visits and meetings. In terms of country-specific findings, Niger demonstrated both Interactive and Political models of research utilization by using iCCM to capitalize on the existing health infrastructure. Both Mozambique and Kenya exhibit Problem-Solving research utilization with different outcomes. Furthermore, the persistent quest for additional evidence suggests a Tactical use of research in Kenya. Results presented here indicate that while evidence from research studies and other contexts can be critical to policy development, local evidence is often needed to answer key policymaker questions. In the end, evidence may not be enough to overcome resistance if the policy is viewed as incompatible with national goals.

Key Messages

- Local data, such as national surveys and health monitoring data, served to define the policy problem of children dying at home but overall policy formulation was influenced by a broader variety of evidence, including research, experiences in other countries, and both local and international pilot projects.
- Local evidence was highly valued by local decision makers and the lack of it inhibited policy progress.
- Development partners brought evidence to policy discussions, especially peer-reviewed publications and guidelines.
- Evidence of interactive, political, problem-solving and tactical models of research utilization was seen in the three study countries.

## Introduction

Integrated Community Case Management of Childhood Illness (iCCM) is a policy that encompasses the treatment of malaria with artemisinin combination therapy (ACT) and other antimalarials, treatment of diarrhoea with low-osmolarity oral rehydration salts (ORS) and zinc and treatment of pneumonia with antibiotics, all provided for children below 5 years by community health workers (CHWs) at the household and/or community level. This policy has evolved in recent years in response to limited success of earlier child survival strategies.

After the Alma Ata conference in 1978 (1978), the ‘child survival revolution’ in global public health advocated for several key interventions that shifted attention towards vertical, disease-specific programmes to combat childhood illness, such as the control of diarrhoeal disease and immunization (Walsh and Warren 1979; van Olmen *et al.* 2012). In 1995, the clinical algorithm for Integrated Management of Childhood Illness (IMCI) was developed to address case management of the most common illnesses for children below 5 years: pneumonia, diarrhoea, malaria, measles and malnutrition (WHO 1997). The IMCI programme was originally articulated as being three pronged: improving (1) facility-based case management, (2) health systems and (3) family and community health practices (WHO 2013); however, the community component was often not successfully implemented in many African countries (Lambrechts *et al.* 1999; WHO 2003; Bryce *et al.* 2005).

The evidence base for the delivery of curative services at the community level for the individual pathologies of diarrhoea, malaria and pneumonia is substantial (Sazawal and Black 1992; 2003; Delacollette *et al.* 1996; Bhutta *et al.* 1999; Kidane and Morrow 2000; Victora *et al.* 2000; Baqui *et al.* 2002; 2004), while the research supporting the ‘integrated’ delivery of these services is more sparse (Lewin *et al.* 2010; Yeboah-Antwi *et al.* 2010; Christopher *et al.* 2011; Chinbuah *et al.* 2012). Despite the paucity of evidence around the integrated delivery of curative services, actors at the global level have taken pains to present iCCM as an evidence-based policy (see Dalglish, George et al. in this issue for further exploration of global-level evidence and the role of global actors) and a series of global-level statements form the basis for iCCM (WHO/UNICEF 2004a,b; 2012).

This article is part of a larger cross-country case study aiming to analyse iCCM policy development using Walt and Gilson’s policy triangle framework (Walt and Gilson 1994). Although the policy triangle framework guided the overall study, we found that it did not sufficiently help explain the differences in evidence use emerging in the study countries and so we reviewed other models specific to evidence use to approach the data with a different lens.

### Models for evidence use in policies

One early approach from the public administration literature to understanding how evidence influenced policies proposed three mechanisms: instrumentally, resulting in direct change; conceptually, enlightening users in unspecific ways or symbolically, to support existing positions (Weiss 1979; Beyer and Trice 1982), although it has been widely acknowledged that evidence is not usually used rationally. The work by Carol Weiss in the field of ‘research utilization’ focused on the use of social science research in policymaking for education policy (Weiss and Bucuvalas 1980; Weiss 1995) and some of her earlier works identified seven models for research utilization that are still applicable today (Weiss 1979). These models range from ones that can be described as ‘rational’, such as the Knowledge-Driven Model, to others that are more one dimensional, such as the Political Model, to fluid, multi-dimensional, more complex models like the Interactive Model (Table 1). These models are not mutually exclusive as any one policy process could fit under more than one rubric at once. Most of the applications of Weiss’ models and subsequent efforts to assess complexity in evidence use have focused on policymaking in high-income countries, especially the US, UK and Canada.

**Table 1:** Models of research utilization

Model	Description
Knowledge-Driven Model	The mere existence of evidence presses towards use. However, little evidence is so compelling as to drive implementation
Problem-Solving Model	Application of research results to pending decisions. Expects that evidence will reduce uncertainty and solve a policy problem for which there are agreed-upon goals. Research may antedate the policy problem or may be commissioned once a problem is identified
Interactive Model	Interactive search for knowledge and research is only a part of a complex process based on experience, politics, pressure and judgment
Political Model (Affinity model)	Research is used to support pre-existing positions around an issue or decision. This is not an illegitimate use of evidence because it can still reduce uncertainty
Tactical Model	What matters is not the content but the fact that research is being done around the issue, with research being used as a delaying tactic or held up as proof of responsiveness to an issue
Enlightenment Model	Concepts and theoretical perspectives resulting from research permeate the policy-making process indirectly. Evidence does not have to be compatible with current thinking and values to be useful but this type of diffusion risks simplification and distortion and is largely inefficient for reaching policymakers
Research as Intellectual Enterprise	Characterized by the interaction between science and policy in response to broader societal changes, with research as one manifestation of this exchange

*Source:* Adapted from Weiss (1979).

Katherine Smith draws from Weiss’ work to study UK policies and has advocated for exploring complexity in policymaking and the different potential mechanisms for evidence writ large to influence policy, including recognizing the variety of actors in this process and the central role of politics as more than a barrier to evidence-informed policy (Smith and Joyce 2012; Smith 2013). There also have been calls from those working in developing countries to start taking into account the broader, largely political environment in which policy decisions are made (Hennink and Stephenson 2005; Gilson and McIntyre 2008; Hyder *et al.* 2011). Oliver *et al.* (2014) also highlight the need to elucidate ‘what’ evidence policymakers use and ‘how’ they use it to answer key questions.

Various researchers have explored how context affects evidence use and how policymakers faced the same issue and evidence interpret and use evidence differently (Dobrow *et al.* 2006; Greenhalgh and Wieringa 2011; Wathen *et al.* 2013), but there are few descriptions of how evidence informs policymaking when comparing the same policy across low- and middle-income country (LMIC) settings (Woelk *et al.* 2009). With this in mind, this article aims to assess if, how and why evidence played a role in policy formulation for iCCM in three LMICs, Niger, Kenya and Mozambique, and categorize the patterns of evidence through Weiss’ seven models.

## Materials and methods

The larger study conducted qualitative retrospective country case studies in Burkina Faso, Kenya, Malawi, Mali, Mozambique and Niger. These countries were selected to represent variability around three policy outcomes of interest, namely: (1) the current stage of iCCM policy and programme development, (2) the level of role expansion required for CHWs to take on iCCM and (3) whether CHWs are paid or are volunteers. Countries were identified based on researcher-developed profiles drawing on discussions with United Nations Children's Fund (UNICEF) and country officials, other researchers work on iCCM and results from an earlier iCCM policy survey (de Sousa *et al.* 2012; George *et al.* 2012). By including six countries that were at different points of the policy cycle and likely facing different levels of barriers to implementing iCCM, the transferability of findings to other contexts is enhanced (Yin 2009).

The methodological approach included a documentary review and in-depth interviews with key stakeholders in the country, which were triangulated to support analysis and corroborate findings (Yin 2009). Potential respondents were identified through the documentary review and snowball sampling of interviewees. A common interview guide based on key concepts from the policy triangle framework (Walt and Gilson 1994) was jointly developed by researchers from the Johns Hopkins Bloomberg School of Public Health (JHSPH) in collaboration with researchers from the study countries. Interviews were conducted in the local language and transcribed without translation.

An analysis plan was developed collaboratively between the team at JHSPH and in-country research teams through in-person workshops following a descriptive content analysis approach (Berg 2004; Hsieh and Shannon 2005). A common study-wide codebook was developed based on the policy triangle framework, with country teams developing additional codes as needed during data analysis. Primary thematic analysis was conducted deductively in each study country using NVivo software (QSR International) with additional support from JHSPH. Further synthesis was conducted to identify cross-cutting themes and these results are available elsewhere (Bennett *et al.* 2014).

For this article, we focus on three countries where iCCM policy development progressed at varying speeds to explore the use of evidence in highly variable contexts: Niger, an early adopter with full implementation of iCCM; Mozambique, where policy development moved more slowly but was adopted; and Kenya, which had yet to adopt a comprehensive iCCM policy. The study was implemented in each country by a local research team, with the exception of Niger where a JHSPH doctoral student took the lead on the research with support from local researchers. Data were collected between January and September 2012. Ethical approval was obtained from JHSPH, Great Lakes University-Kisumu in Kenya and the National Bioethics Committees in Mozambique and Niger.

## Results

The results from the three study countries presented here represent 204 documents and a total of 72 interviews (Table 2), with combined analysis of individual country case reports and the additional cross-country synthesis. For each country, a brief paragraph introduces the local background followed by descriptions of how evidence was used in policymaking, including types of evidence used.

**Table 2:** Documents and interviews informing these findings

	Niger	Kenya	Mozambique	Total
Number of documents reviewed	113	41	50	204
Interviews conducted by category				
Government officials, incl. Ministry of Health and other government ministries	18	10	8	36
Multilateral agencies, e.g. UNICEF, WHO	8	3	5	16
Donors and bilateral agencies, e.g. USAID, CIDA	3	1	1	5
NGOs, incl. national and international	2	3	5	10
Other actors, incl. civil society, researchers, professional associations, etc.	1	2	2	5
Total respondents interviewed/approached	32/37	19/31	21/40	72/108

### Niger

Although over 80% of the Nigerien population has always been rural, health services in Niger have historically been concentrated in cities; the problem of limited access to health care was thus well-known to policymakers in the 1990s and early 2000s (Körling 2011). Ongoing high rates of child mortality were also known to policymakers through demographic and epidemiological data, mainly the Demographic and Health Surveys (Kourguéni *et al.* 1993; Attama *et al.* 1999; Institut National de la Statistique and Macro International Inc. 2007) and data from the National Health Information System.

In 1997, Niger became one of the first Francophone countries to adopt IMCI, and between 2001 and 2010 over 2000 health huts were built in rural areas (Oliphant *et al.* 2011). However, the effect of IMCI was limited mainly to the urban health system, particularly as the community-level component (C-IMCI) was barely implemented: in 2007, only 10 of the 42 health districts had begun implementing C-IMCI (Hamsatou 2008; Tawfik *et al.* 2001).

Starting in 2001, Mamadou Tandja—then president of Niger—created a nationwide network of health huts financed through funds from Heavily Indebted Poor Country initiative (HIPC). Later, in 2006, a policy promulgated by President Tandja guaranteed free health care to children below 5 years, and significantly increased health centre attendance (Ousseini 2011; Dalglish, Surkan *et al.* this issue). The huts were staffed by a new cadre of CHWs based at health posts. These CHWs have a middle school education and receive 6 months of training with 1 week targeted for iCCM. They are paid a salary ($100/month) through HIPC funds, and therefore are not entirely integrated into the health system infrastructure (Bennett *et al.* 2014).

As the deadline approached for the Millennium Development Goals (MDGs), pressure increased to make progress on child survival rates and indeed MDG 4 is mentioned in almost all iCCM documents, and progress towards all the MDGs was regularly reported on by the National Institute of Statistics in Niger (MEF and ONU 2004; Institut National de la Statistique 2007; 2010).

The CHW cadre that was already in place paired with the network of health huts that had been established during President Tandja’s tenure allowed for iCCM to be viewed as a viable solution that built on the existing health infrastructure in place and extend the reach of the health system.

International partners played a key role in raising awareness and interest in iCCM as a policy option, largely by sharing evidence of potential interventions with Nigerien policymakers. One primary way they did so was by organizing regional meetings and supporting Nigerien stakeholders’ attendance. A regional meeting in Dakar in 2005 sponsored by United States Agency for International Development (USAID) was especially influential as Senegalese participants shared their experiences with community management of acute respiratory infections.

“They went to Senegal, they saw the Senegalese experience, they came back here and we worked in the Madarounfa district [and] trained some people for the screening and diagnosis of ARIs.” (Niger—Development Partner 024)

“We took the scientific evidence, for instance ARIs in Senegal. This is really the foundation. Also surveys from Pakistan, and India and WHO and USAID were targeting Benin and some other countries … There was scientific evidence indicating the basis for the management of respiratory conditions, and therefore combined with the other conditions, this is iCCM …” (Niger—Development Partner 003)

International partners also shared evidence on iCCM from scientific journals, specifically the 2003 ‘Lancet’ series on child survival (Black *et al.* 2003; Bryce *et al.* 2003; Claeson *et al.* 2003; Jones *et al.* 2003; Lee 2003; Venis 2003; Victora *et al.* 2003). Local stakeholders’ view was that if guidelines or evidence were brought forth by internationally respected partners, then the need for discussion was minimal because it already had the imprimatur of legitimacy. All that remained was to fit the recommendations to the Nigerien context.

“The role of evidence is that there is no point reinventing the wheel. These are things that are immediately applicable because they have proven their worth. They do not need to be tested anymore and this makes you move faster to achieve a reduction in child mortality and morbidity.” (Niger—Clinician 007)

“WHO enjoys great trust. They have a lot of confidence in WHO directives. We present the directives and later they adapt [them] …” (Niger—Development Partner 006)

Although the totality of the evidence was seen as influential in its own right, there was still a need to conduct a local pilot project to demonstrate feasibility. The pilot was viewed as a necessary precursor to large-scale implementation to adapt iCCM to the local context; it was conducted in Madarounfa in 2005 with community volunteers—not CHWs—emulating the project in Senegal.

“This is why we proposed to move ahead as a pilot project in order to truly understand the program. All the difficulties would be identified and after the final report, we would see how to expand it to the entire country without having much difficulty … [that is] why we opted for a pilot program.” (Niger—Government official 008)

The pilot was refocused to work with CHWs in 2006 once it was realized that community volunteers could not legally handle antibiotics. A mid-term evaluation of the Madarounfa pilot was released in 2007 indicating positive results and the decision was rapidly made to scale up iCCM nationwide. iCCM policy was adopted in 2008 (MSP 2008).

### Kenya

Kenya’s health system is better resourced than many other countries in sub-Saharan Africa with 

0.93 health workers per 1000 population, including 0.05 physicians and 0.41 nurses and midwives per 1000, respectively (Africa Health Workforce Observatory 2010), and Int$140 per capita spent on health (Countdown to 2015: Maternal Newborn and Child Survival 2012). CHWs have historically been trained by non-governmental organizations (NGOs), although the Ministry of Health had recently begun to formalize CHWs’ role in the health sector through its community strategy (Ministry of Health 2007). CHWs receive 2–6 weeks training with 1 week targeted for iCCM, and are recommended to have basic literacy skills and receive payment ($24/month) when funds are available, but this is unevenly practiced (Bennett *et al.* 2014).

Kenya has had policies and programmes on home management of malaria including treatment, and the use of ORS for diarrhoea (MOPHS 2009; 2010). There has been resistance to including zinc for diarrhoea due to concerns about side effects and although the most recent policy for treatment of childhood diarrhoea includes ORS and zinc, there is no clear guidance on whether these can be used by CHWs or when it will be available in the CHW supply kit (MOPHS 2010). There has also been continued debate about antibiotic treatment for pneumonia at the community with local decision makers repeatedly articulating a need for more evidence.

As in Niger, policymakers were aware of challenges to access health-care services at the community level as identified in various policy documents, a DHS survey (Ministry of Health 2005) and an IMCI evaluation (Mulei *et al.* 2008). These data, combined with pressure to meet the MDGs, put iCCM on the governmental agenda.

“Looking at the countries’ data, you find that almost 50% of the children do not access the facilities. So that means that quite a number of children are missing health interventions at the community level. We don’t want them to miss out in this treatment intervention; we want to take interventions near to them so that we can reduce the deaths due to severe diseases and eventually have an impact on reducing mortality rates.” (Kenya—NGO 014)

International partners were a main source of evidence in this case as well. The 2003 Lancet series on child survival was shared at stakeholder meetings, and UNICEF sponsored a study tour for Ministry of Health officials in 2009 to Malawi, Ethiopia and India to see iCCM policies in action. WHO also organized a meeting in Nairobi in 2011 on ‘coordinated approaches to pneumonia and diarrhea prevention and control’ (WHO 2011), which was seen as a mechanism to pressure the Ministry to adopt a policy that included pneumonia treatment.

“I would say it has been consultative enough, the government was not initially ready for it despite the overwhelming evidence that iCCM works. But through advocacy and push from international organizations the government is now supporting it. We see the big role of non-state actors in influencing the process, particularly WHO and UNICEF. Left to government alone it would have taken much longer.” (Kenya—NGO 019)

Kenyan Ministry of Health decision makers questioned the applicability of the evidence from other settings that was being shared with them. In particular, they questioned whether iCCM was an appropriate policy for their country given the structure of their health system, the composition of their CHW cadre and the greater availability of nurses who could be delivering these services.

“Yes … there were some citations that were made from I think Cuba where similar iCCM activities had happened, they cited also Malawi which I disputed because I know how Malawi looks like, the iCCM is not by CHWs, they mistake the people they normally call health surveillance assistants … the picture for Kenya is very different … because they [others] are community members but not volunteers. They are all in government pay roll.” (Kenya—Government Official 007)

There have been a number of efforts in Kenya over the years to pilot community-level delivery of treatment but with limited success. An early trial started by CARE in the Siaya district in the late 1990s trained CHWs to use a simplified IMCI algorithm for treating diarrhoea, malaria and pneumonia. However, the results indicated that CHWs were not treating children correctly (Kelly *et al.* 2001) and adherence to treatment guidelines declined over time, even with refresher courses (Rowe *et al.* 2007a, b). A local pilot study of community IMCI had been conducted by Catholic Relief Services in 2003 in Mbeere district which indicated that CHWs could diagnose and treat malaria, but they should only be allowed to recognize and refer for pneumonia. In 2011, ‘Save the Children’—Kenya implemented an iCCM project in Wajir but they were not granted permission to use antibiotics.

Given these unconvincing experiences with local pilots for pneumonia care, treatment with antibiotics is still being debated by policymakers as they deem existing evidence to be insufficient. Kenyan stakeholders felt that new pneumonia-specific pilots were needed before making a decision on the policy. Two pilots focused on antibiotic treatment for pneumonia at the community level were commissioned by WHO and its partners and carried out by Kenya Medical Research Institute (KEMRI) and AMREF and were ongoing at the time of this study.

“There is need for evidence for policy makers. They continue asking how do we do it? Now that’s why we are doing the research. We are still moving on. Integration in CHW training manual has to be within the framework. We worked closely within community strategy … but they wanted more evidence.” (Kenya—Multilateral Organization 018)

“For pneumonia (pauses) I think there is a long way to go, there are discussions that are ongoing at the moment to look at possibilities of whether we can actually introduce treatment of pneumonia at community level and what the government has, at that level, to give us evidence or how it would work for that matter so for me- pneumonia has longer way to go than any of the other two.” (Kenya—Donor/Bilateral Organization 005)

As described, iCCM policy development has been more contentious in Kenya (see Juma *et al.* this issue). Commitment to the overall policy is unclear and there was a general feeling across respondent groups that iCCM was being pushed by external partners. Kenya has reached consensus on some aspects of iCCM but these are only present in training guidelines for CHWs which had been drafted in 2011 and were finalized in 2013 (electronic versions are not available yet) (Ministry of Public Health and Sanitation 2011), not in more significant policy documents as yet. Although pilots for iCCM are planned and intended to inform broader programme development and implementation, no direct funding for the programme has been allocated by the government.

### Mozambique

In Mozambique, there was strong political commitment to the CHW revitalization policy (Ministério da Saúde 2010), which was the overarching policy under which iCCM would be included, so the need for evidence to convince policymakers was not as strong (see Chilundo *et al.* this issue). The revitalization of CHWs, known as ‘Agentes Polivalentes Elementares’ or APEs, was seen as a critical way through which to institutionalize the government’s commitment to community involvement in addressing health issues. As such, CHWs are seen as part of the Ministry of Health and their activities are integrated into the health system (MISAU 2004). CHWs have minimal literacy and arithmetic skills, receive 14 weeks training with 4 weeks targeted to iCCM and they are supposed to receive a monthly stipend ($43/month) (Bennett *et al.* 2014).

Like in the other study countries, large-scale surveys identified access as a significant cause of child mortality and the commitment to meet the MDGs put pressure on Mozambican policymakers to advance policies and programmes that would address this issue (INE and Macro International Inc 1998; INE, MISAU and USAID 2005; MISAU, WHO, UNFPA, USAID/FORTE SAÚDE, UNICEF and PATHFINDER 2008; INE, MISAU and UNICEF 2009; INE 2012). This was intensified by reports indicating that Mozambique was one of the few countries on track to meet MDG 4 (República de Moçambique 2010) and should continue its work to meet targets.

“… before launching the revitalization [of the CHW program], it was commissioned a study … to assess the situation of community health in Mozambique… it was on the basis of this study that the Ministry realized the need to do something …” (Mozambique—NGO 018)

There had been a long history of CHWs treating malaria and diarrhoea at the community level, and of using evidence to update the recommendations for CHW-delivered treatment before the advent of iCCM as a self-contained policy. The policy regarding medications to treat malaria was updated in 2011 based on evidence from international studies as well as local studies (MISAU 2011). In response to increasing evidence of resistance to sulphadoxine–pyrimethamine (SP), a study was conducted in Maputo in 2002 using SP and artesunate at health facility and community levels (Lubombo Spatial Development Initiative 2003). This study coupled with WHO recommendations (WHO/TDR and Roll Back Malaria 2004) resulted in the adoption of ACT for delivery by CHWs in 2004. Likewise, diarrhoea treatment was updated with help from local evidence in 2010 (MISAU and DNSP 2010).

The National Policy of Infant and Neonatal Health in 2006 (MISAU 2006) proposed iCCM and the revitalization of the CHW programme, but the policy of revitalization itself, which details implementation of iCCM, was not approved until 2010 (Ministério da Saúde 2010). During the 4-year lag between the proposal and approval of the policy, various actors played a role in introducing evidence into the policy process.

UNICEF, WHO and international NGOs shared evidence from guidelines, research and experiences of other countries, including Zambia, Malawi, Uganda and from Latin America, supporting the delivery of antibiotics by CHWs in order to advocate for the introduction of antibiotics for pneumonia which was facing resistance. The advocacy went so far as to push for amoxicillin instead of cotrimoxazole.

“… but it was more due to advocacy to change to dispersible amoxicillin, because it was recommended by WHO … it was more local advocacy from Save the Children, Malaria Consortium and UNICEF …” (Mozambique—Bilateral organization 004)

“… [these] were studies that were done using community workers—this figure in the community—trained to use antibiotics in case of pneumonia or zinc, ORS in the case of diarrhea that could get good results. Then there starts to be a lot of disseminating results in Africa, in Kenya, in Uganda, then countries that do not adhere to policies recommended by WHO and UNICEF would be countries who would be at the margin of others who want to achieve the millennium goals. Often therapeutic policies go against policies, in other words the external pressure is very strong to the point of changing the own national treatment policy … we suddenly see that things are being changed but it is due to external pressure.” (Mozambique—NGO 018)

International partners helped generate local evidence to support different iCCM components. Likewise, in 2009 a local study supported by WHO and USAID and carried out by the National Institute of Health evaluated the potential for including zinc in the community strategy for treating childhood diarrhoea (Food for the Hungry 2009). The findings from that pilot were used directly to advocate for a change to the medications in the kits CHWs carry.

“… there is a study in Mozambique on zinc conducted by Dr. Mbofana in Beira, which we used to influence the pharmacy staff in order to change the content of Kit C within the new APE criteria …” (Mozambique—Multilateral Organization 015)

In 2007, Paulo Ivo Garrido—Minister of Health at the time—found the 2003 Lancet series on child survival so convincing that he had the articles translated into Portuguese and disseminated for discussion during a key meeting on community involvement demonstrating high-level political commitment to iCCM-related strategies. His support helped finalize the inclusion of iCCM activities in the CHW policy.

“… do you know who sent the Lancet series to translate? … Ivo Garrido received [it], liked … the Lancet opened his eyes to … neonatal … and … zinc for diarrhea cases at the primary level … all this reduces a good percentage of infant mortality …” (Mozambique—Government Official 006)

## Discussion

The case studies of Niger, Kenya and Mozambique present three varying experiences with evidence. As we map these case studies to Weiss’ models of utilization, we see different dynamics at work. The interplay between different forces in Niger suggests an Interactive model of research utilization. However, the opportunity for policymakers to use iCCM to capitalize on existing investments in health huts and CHWs also indicates a Political model. The use of evidence in Mozambique was fairly straightforward and aimed at answering specific policy-relevant questions, such as a project piloting zinc for diarrhoea treatment. This kind of use is most aligned with the Problem-Solving model and also reflects the historical use of evidence to inform child survival policies in Mozambique. In Kenya, there seem to be two models at work. First, the resistance to community-level pneumonia treatment by CHWs was not overcome by existing evidence, including local pilot projects that showed limited success, suggesting that policymakers used a Problem-Solving approach by ‘not’ adopting community pneumonia treatment. Furthermore, the broader resistance to CHWs providing clinical care and the lack of financial commitment calls into question the overall support that Kenyan policymakers have for an overarching iCCM policy. Thus, their demands for more and more evidence may actually be a stalling technique to postpone a final decision suggesting a Tactical use of research.

Linking Weiss’ models with the policy triangle framework, we see that although all three countries identified child mortality as a key issue, the role of evidence in defining a policy solution was murkier (process) depending on the commitment to iCCM as a potential policy (issue), the local health system infrastructure (context) and the interplay between national and international stakeholders in sharing and pushing evidence (actors). Unsurprisingly, the value of evidence varied by context but there are several lessons that can be drawn from this study regarding how policymakers used evidence.

First, different types of evidence had specific usefulness in the policymaking process. National monitoring data, such as surveys, programme evaluations and service utilization data, were essential in recognizing the policy issue at hand, and highlighting issues around access to health services and that children were ‘dying at home’ before reaching facilities; on this point there was consensus across stakeholders in each country. Research evidence was more crucial to identifying policy options, specifically community-level interventions, but was also more likely to be debated, with local pilot projects playing a critical role in all study countries in different ways. The Lancet series on child survival was widely recognized across settings to have played a major role as a source of evidence. This series was being used and referenced several years after its publication though interestingly, it appears that respondents ascribed to the series a message that it did not directly make: that child survival interventions should be delivered at the community level. This misconception could have resulted for a number of reasons. It is possible that readers simply misconstrued and/or extrapolated the findings. Alternatively, because the series was shared with national policymakers through international partners, it is also possible that the message was mediated and modified either to justify a more community-oriented approach or because in the intervening years the field had moved beyond the series’ original message. Unfortunately, our study could not determine the main reason underlying this shift in interpretation of the series’ message.

Second, it is critical to acknowledge the role of development partners in introducing evidence in iCCM policy discussions, especially UNICEF and WHO (see Bennett, Dalglish *et al.* this issue). These actors functioned largely as knowledge brokers by sharing research studies regarding iCCM interventions, sharing other countries’ experiences with iCCM, and sponsoring and supporting site visits to other countries and regional meetings. UNICEF and WHO’s role as trusted actors in the policy arena provided them and their technical assistance with good standing among local actors. By virtue of their position and mandate, these agencies have access to evidence from a variety of sources that they can then ‘digest’ and streamline to bring to bear during policy discussions. Walt *et al.* (2004) have suggested an iterative transfer loop of evidence between national and global actors starting with knowledge generation at the national level followed by policy consolidation and standardization at the international level, and a third loop focused on policy marketing and promotion from the international level to national actors, leading to oversimplification of complex interventions. To some extent, this reflects the experience with iCCM: the initial evidence for community-based treatment was generated in South Asia followed by best practice policies developed at the global level (Dalglish, George *et al.* this issue) and promoted at the national level through advocacy, joint statements and guidelines and study trips.

Third, there was an interesting interplay in these cases between local and international evidence. For some national-level actors, there was an implicit trust that the advice that international partners provide, whether through informal advice or formal guidelines, was based on evidence and could be trusted while at the same time, the need for pilot projects to either test or legitimize the interventions precluded indiscriminate adoption. Policymakers’ need for local evidence before embarking on policy reform suggests a sceptical approach to wholesale importation of evidence from other contexts, even when committed to the overall policy objectives. In another cross-country policy analysis, Woelk *et al.* (2009) compared the policymaking around the introduction of magnesium sulphate for eclampsia and the use of insecticide-treated nets and indoor residual household spraying for malaria in Mozambique, South Africa and Zimbabwe. They found that policymaking around nets and spraying used varied types of evidence, with a greater demand for local evidence (Woelk *et al.* 2009). They propose that, unlike clinical interventions, public health interventions require more evidence on implementation and sustainability, and local evidence is seen as more credible (Woelk *et al.* 2009). Burchett *et al.* (2013) report similar findings from Ghana regarding the applicability and transferability of ‘foreign’ research which found that when stakeholders judged evidence from other settings, they were most concerned by the implementability of the intervention rather than its potential effectiveness. Our findings about iCCM and the need for local evidence, especially around implementation, echo these ideas—even in Niger, the most iCCM friendly case in this group, a pilot study for iCCM was seen as necessary to prove the feasibility of the intervention in the Nigerien context despite its success in nearby Senegal. Future research should further explore how the type of intervention and supporting evidence influence policymaking, especially for interventions where global stakeholders like UN agencies, funders and advocates are closely tied to norm setting.

More and more, policymakers are being pushed to base their decisions on evidence that is regularly evolving and, occasionally, changing, especially for complex interventions that cannot be tested everywhere prior to adoption. In that light, we would be remiss in not reporting recent evidence around iCCM since the completion of country studies, some of which have not been as supportive. A 2012 supplement in the ‘American Journal of Tropical Medicine and Hygiene’ presented results from iCCM programmes across Africa. iCCM programmes were generally associated with increased access to care but treatment rates often varied by disease type and severity (Cardemil *et al.* 2012; Chinbuah *et al.* 2012; Lainez *et al.* 2012; Mukanga *et al.* 2012; Nsona *et al.* 2012; Rutebemberwa *et al.* 2012; Seidenberg *et al.* 2012) and consistent, accurate treatment can be a challenge (Cardemil *et al.* 2012; Mukanga *et al.* 2012), especially in the absence of regular, effective supervision (Lainez *et al.* 2012). Furthermore, a recent review called into question how well community-level treatment of childhood pneumonia can work in sub-Saharan Africa, whether delivered singly or in an integrated fashion (Druetz *et al.* 2015). Some of the challenges identified are directly linked to characteristics of sub-Saharan African countries in contrast with the countries that served as models for iCCM and its components, namely India and Nepal, such as CHW structures or being malaria endemic (Druetz *et al.* 2015). These latter issues in particular reflect the questions being asked in our study countries, namely Kenya, about the cross-setting transferability of iCCM evidence.

Finally, we would like to reflect on the frameworks that guided this study. The simplicity of Walt and Gilson’s policy triangle is an excellent entry point for policy analysis across contexts; however, its simplicity is limiting and, in fact, it was not intended to address evidence use directly. Given the early stage of research analysing the use of evidence in LMICs, especially across contexts, we felt that Weiss’ framework provided a useful analytical tool to supplement the policy triangle in our analyses. Although Weiss’ work has been used in public policy analyses, even in health, this is the first application of her models of utilization in LMIC contexts. We felt that this public policy framework, which presents broad, overlapping categorizations, was an ideal candidate to explore which potential models of evidence use were present. What we are unable to discern is ‘why’ the experiences differed in the three countries profiled here. If we can say that the policy was the same and the majority of the actors (in terms of institutions, not individuals) were the same, does it all fall to the specific context and the process of policy development? Further research is needed to explore these distinctions more deeply.

There are several limitations to this study. First, this cross-country analysis drew primarily from the individual country reports and the synthesis report with limited examination of primary data sources. Although we are confident that the individual country reports were thorough, it is possible that some details were missed given the need to synthesize data sources to present a cohesive picture, but we mitigated this by going back to country case study authors and original data. Second, the data collection for these case studies took place between 2 and 4 years after formal policy adoption and even more distantly from policy formulation, which likely affected the recall of our respondents. We addressed this by interviewing a variety of stakeholders involved in policy development but also triangulating our findings with the documentary review.

## Conclusion

As global-level initiatives are translated into national settings, it is important to recognize that international evidence may be insufficiently convincing; more importantly, national policymakers may make an evidence-informed decision by determining that a specific policy should not be implemented in their country. It is clear that public health policies require multiple sources of evidence, especially local data, highlighting the need for targeted funding aimed at answering key questions posed by local policymakers regarding implementability and sustainability. Efforts of this nature have begun (Wazny *et al.* 2014) and should continue.
